# Three Water Restriction Schedules Used in Rodent Behavioral Tasks Transiently Impair Growth and Differentially Evoke a Stress Hormone Response without Causing Dehydration

**DOI:** 10.1523/ENEURO.0424-21.2021

**Published:** 2021-12-07

**Authors:** Dmitrii Vasilev, Daniel Havel, Simone Liebscher, Silvia Slesiona-Kuenzel, Nikos K. Logothetis, Katja Schenke-Layland, Nelson K. Totah

**Affiliations:** 1 Helsinki Institute of Life Science (HiLIFE), University of Helsinki, Helsinki 00790, Finland; 2 Department of Physiology of Cognitive Processes, Max Planck Institute for Biological Cybernetics, Tübingen 72076, Germany; 3 Zentrale Forschungs Einrichtung, Goethe Universität, Frankfurt am Main 60323, Germany; 4 Institute of Biomedical Engineering, Department for Medical Technologies and Regenerative Medicine, Eberhard Karls University Tübingen, Tübingen 72074, Germany; 5 Division of Imaging Science and Biomedical Engineering, University of Manchester, Manchester, M13 9PL, United Kingdom; 6 NMI Natural and Medical Sciences Institute at the University of Tübingen, Reutlingen 72770, Germany; 7 Department of Medicine/Cardiology, Cardiovascular Research Laboratories, David Geffen School of Medicine, University of California Los Angeles, Los Angeles 90095, CA; 8 Faculty of Pharmacy, University of Helsinki, Helsinki 00790, Finland

## Abstract

Water restriction is commonly used to motivate rodents to perform behavioral tasks; however, its effects on hydration and stress hormone levels are unknown. Here, we report daily body weight and bi-weekly packed red blood cell volume and corticosterone (CORT) in adult male rats across 80 days for three commonly used water restriction schedules. We also assessed renal adaptation to water restriction using postmortem histologic evaluation of renal medulla. A control group received *ad libitum* water. After one week of water restriction, rats on all restriction schedules resumed similar levels of growth relative to the control group. Normal hydration was observed, and water restriction did not drive renal adaptation. An intermittent restriction schedule was associated with an increase in CORT relative to the control group. However, intermittent restriction evokes a stress response which could affect behavioral and neurobiological results. Our results also suggest that stable motivation in behavioral tasks may only be achieved after one week of restriction.

## Significance Statement

Neuroscience research has seen the growing use of water restriction in studies using head-fixed rodents. Despite this growing use, the effects of various water restriction schedules on hydration and stress hormone levels are unknown. Here, we assess hematocrit (Hct) and blood corticosterone (CORT) over 80 days and compare three commonly used restriction schedules. Our results show that one type of restriction schedule evokes a stress response, which may have unanticipated neurobiological and behavioral consequences.

## Introduction

The use of water restriction to motivate rodents to perform goal-directed behavioral tasks has expanded in recent years because of the adoption of head-fixed behavioral paradigms using a water licking spout (Schwarz et al., 2010; Lee et al., 2012; Polack et al., 2013; Sachidhanandam et al., 2013; Scott et al., 2013; Reimer et al., 2014; Zhang et al., 2014; McGinley et al., 2015; Yttri and Dudman, 2016; Jurjut et al., 2017; Mathis et al., 2017; Runyan et al., 2017; Steinmetz et al., 2019; Stringer et al., 2019; Schröder et al., 2020; Foo et al., 2021; Peters et al., 2021). Head-fixation has opened up new avenues of research in rodents, which were heretofore conducted largely in non-human primates, such as studies on visual perception (Khastkhodaei et al., 2016; Sriram et al., 2016; Pakan et al., 2018; Jacobs et al., 2020; Jin and Glickfeld, 2020; International Brain Laboratory et al., 2021), forelimb reaching (Yttri and Dudman, 2016; Mathis et al., 2017; Galiñanes et al., 2018), and arousal (using pupillometry; Reimer et al., 2014, 2016; McGinley et al., 2015; Vinck et al., 2015). Additionally, there has been a growing interest in using head-fixed rodents to study the neural representation of space using virtual reality (Harvey et al., 2009; Radvansky and Dombeck, 2018). Head-fixation of rodents has also been used to gain access to membrane potentials during goal-directed behavior (Polack et al., 2013; Sachidhanandam et al., 2013; McGinley et al., 2015). Thus, the use of water restriction as a motivational tool for rodent behavioral paradigms will likely continue to be a fundamental tool in neuroscience.

However, the results of behavioral and neurobiological studies could be affected by the stress of water restriction, which has not been assessed in any of the water restriction schedules that are commonly used in neuroscience research. One study using rats limited access to water at 30 min/d and reported significant elevation of blood plasma ACTH and adrenal corticosterone (CORT) after 6 d (Arnhold et al., 2009). Training and measuring behavior and recording neuronal activity, on the other hand, can last many weeks of months and require water restriction well beyond a 6 d (Schwarz et al., 2010; Scott et al., 2013; McGinley et al., 2015; Yttri and Dudman, 2016; Mathis et al., 2017; Runyan et al., 2017; Jin and Glickfeld, 2020; International Brain Laboratory et al., 2021). Another study found no elevation of plasma CORT after 37 d during which rats were provided daily access to water for 15 min (Heiderstadt et al., 2000b). These data suggest that the stress response may adapt during chronic water restriction at some point after 6 d. These studies have provided “snapshots” of the stress response at 6 and 37 d, but the change in CORT over the time course of a typical behavioral experiment remains unknown. Furthermore, these snapshots are limited to one type of restriction schedule, which limited water availability to unlimited volume consumption within a daily time window. To our knowledge, prior studies have not characterized the stress response to other types of schedules used in behavioral neuroscience, such as those that limit the total volume of water available per day. A clear picture of the stress response to the various water restriction schedules used in neuroscience research will enable the field to consider whether behavioral and neurobiological results might be affected by a stress response to water restriction.

The effect of water restriction schedules on hydration is also not well studied. The standard monitoring for dehydration in rodent behavioral neuroscience involves measuring reductions in body weight (Schwarz et al., 2010; Guo et al., 2014; Reinagel, 2018; Urai et al., 2021). Importantly, body weight loss is not an ideal indicator of dehydration in rodents because their adaptive response to water scarcity is mild anorexia; by reducing food volume in the gastrointestinal tract, rodents reduce water lost through feces (Watts, 1999; Desai et al., 2005; Rowland, 2007; Bekkevold et al., 2013). Another standard assessment for dehydration is skin turgor (Guo et al., 2014). Although turgor is easy to deploy and offers a rapid clinical judgment of dehydration, it is subjective and only visible in stages of advanced dehydration. On the other hand, packed red blood cell volume (hematocrit; Hct) may be an objective clinical sign of dehydration (Dorrington Keith, 1981; Hansen and DeFrancesco, 2002). A prior study has shown that Hct in rats was elevated (indicative of dehydration) after 6 d of 30 min of daily water access (Arnhold et al., 2009). It remains unknown whether Hct is elevated during the chronic restriction that is used in behavioral tasks or whether Hct differs according to type of restriction schedule.

Here, we measured daily body weight and bi-weekly plasma CORT and Hct over 80 d in four groups of rats subjected to different water restriction schedules that are commonly used in behavioral studies (Barnet et al., 1997; Denniston et al., 1998; Laraway et al., 2003; Fujisawa et al., 2008; Schwarz et al., 2010; Jaramillo and Zador, 2011; Pan et al., 2013; Scott et al., 2013; Guo et al., 2014; McGinley et al., 2015; Panigrahi et al., 2015; Yttri and Dudman, 2016; Mathis et al., 2017; Runyan et al., 2017; Jin and Glickfeld, 2020; International Brain Laboratory et al., 2021). The restriction schedules were either *ad libitum* availability (control group), continuous volume-limited water, intermittent volume-limited water (i.e., alternating between 5 d of volume-limited daily water and 2 d of *ad libitum* access), or 30-min time-limited water. We found no evidence for dehydration or excessive stress response; however, the intermittent restriction schedule evoked a small stress response. We observed a two-week adaptation period in which body weight is diminished in all three restriction groups and followed by normal growth. Kidney histology was used to measure changes in the renal medulla and demonstrated that these restriction schedules were not severe enough to drive long-term adaptation of the renal system. Overall, we found that months-long use of common restriction schedules in rats maintains rodent welfare in continuous and timed restriction schedules, but that the intermittent schedule evokes a stress response which could affect welfare and potentially affect behavioral and neurobiological outcomes.

## Materials and Methods

### Subjects

Experiments were conducted with 24 male Sprague Dawley rats (specific pathogen free, Charles River Laboratories). The initial weight of the rats was 270.3 ± 2.8 g (SEM). Rats were single housed to control water administration. Rats were housed in IVC cages (1862 cm^2^ floor space and height of 38 cm, GR1800, Techniplast). An 8 A.M.-to-8 P.M. lights on cycle was used so that data could be conducted under normal lighting for the researchers. All experiments were conducted with approval from the local authorities and in compliance with the animal care committee’s regulations.

### Water restriction procedures

Rats were divided into four groups covering three different water restriction schedules and an *ad libitum* control group. The restriction schedules were “timed,” “continuous,” and “intermittent.” The timed group was given 30 min of *ad libitum* access to water each day. The continuous group received ∼12 ml/d. This small volume of water was delivered using a custom-made water bottle that released water only during consumption. If a rat in the continuous group lost >15% of their body weight in a week, then their daily water was increased by 2 ml. The intermittent group received a repeating schedule of 12 ml of water per day for 5 d, followed by 2 d of *ad libitum* water.

The 12-ml volume of water for the continuous and intermittent groups was chosen based on our experience motivating behavioral task performance by head-fixed rats, as well as the physiological needs of adult male rats. Typical water ingestion patterns of the adult (300–400 g) male rat consist of consuming 20–30 ml/d when it is freely (*ad libitum*) available (Toth and Gardiner, 2000; Schwarz et al., 2010). However, rodents have highly effective renal mechanisms for water conservation, which allow them to remain hydrated and healthy when consuming <20–30 ml/d. For example, under conditions in which rats were allowed to consume as much water as they would like in their home cage, but requiring them to perform physical effort for access by pressing a lever, their daily water consumption was lower (15 ml) per day (Nicholaidis and Rowland, 1974). This daily amount (15 ml consumed by 300-g rats) is approximately equivalent to the requirement to prevent cellular dehydration derived from fluid maintenance formulas, which calculate a requirement of 50 ml/kg of body weight per day to maintain normal hydration (Toth and Gardiner, 2000). Further reductions below ∼15 ml/d for a 300-g rat will activate renal mechanisms allowing rats to conserve water and remain hydrated; therefore, 50 ml/kg/d reflects an upper limit to the amount of water that must be allocated on a water restriction schedule. Water restriction protocols are generally designed to reduce water availability below this upper limit because increased water restriction is associated with higher goal-directed behavioral task performance as measured by percent correct choices in a sensory stimulus discrimination task (Guo et al., 2014). In addition to the observation of this phenomenon by Guo and colleagues, we have also observed in our own unpublished head-fixed rat behavioral experiments that rats are not motivated unless they receive 60–80% of this upper limit (i.e., 9- to 12-ml total water per day). For example, we have observed that rats who receive 14–17 ml will, in the next behavior session, omit (not perform) a large proportion of trials (6–33%). Therefore, providing too much water will reduce their motivation and they will not perform the task. Thus, we have found that 10 ml is adequate for most animals to be motivated to perform the task, but some animals must receive only 8 ml. It is possible that rats who require only 8 ml of water per day to perform the task may have stronger physiological mechanisms for water conservation. In general, rats consume 3–8 ml during the behavioral task and the remaining amount (up to 8–12 ml) is provided in the cage. Therefore, for the present experiments, we chose to test 12 ml of daily water allotment.

### Method of blood sampling and measurement of CORT and Hct

A small blood sample (∼0.25 ml) was taken from the tail vein without anesthesia while the rat was held in a restraint tube. Samples were collected bi-weekly. During two weeks before starting water restriction, the animals were handled and habituated to restraint to reduce the stress response during data collection. Blood samples for Hct measurement were collected in a capillary tube and immediately centrifuged. The packed cell volume was measured against a chart calibrated for the capillary. Blood was centrifuged and the blood plasma was harvested and stored at −8°C. Blood plasma CORT was measured by a commercial firm using ELISA kits (Idexx Laboratories).

### Kidney histology

Rat kidneys were freshly fixed in 4% PFA, washed in RNase free water and transferred in 70% RNase free ethanol. Kidneys were bisected longitudinal before automated embedding in paraffin using a STP120 (Thermo Fisher Scientific). Each paraffin-embedded half was sectioned (10-μm sections) using a microtome HM340E (Thermo Fisher Scientific).

Histologic staining was performed on deparaffinized and hydrated serial sections of rat kidney. Hematoxylin and eosin (H&E) staining visualized cell nuclei (black, dark blue) and counterstains cytoplasm and connective tissue fibers (different shades of pink). In detail (also shown in Table 1), staining was started by deparaffinization with two steps of absolute xylene followed by rehydration steps with a descending ethanol row. Hematoxylin staining was done with Mayer’s hematoxylin solution (Carl Roth GmbH, T865.1) for 10 min followed by 10 min bluing in lukewarm running tap water. Counterstain was done with 1% Eosin Y solution (Carl Roth GmbH, 3137.2) for 2 min followed by a differentiation step in 70% ethanol for 30 s. Stained sections were mounted with Roti-Histokitt (Carl Roth GmbH, 6638.1). Stained sections were stored at room temperature until imaging analysis was performed.

**Table 1:** Deparaffinization, rehydration, and H&E staining procedure

Steps in H&E staining procedure		
10 min	Xylene I	Deparaffinization
10 min	Xylene II	
5 min	Ethanol absolute I	Rehydration
5 min	Ethanol absolute II	
5 min	Ethanol 96% I	
5 min	Ethanol 96% II	
5 min	Ethanol 70% I	
5 min	Ethanol 70% II	
5 min	Ethanol 50% I	
5 min	Ethanol 50% II	
5 min	Distilled water	
10 min	Mayer’s hematoxylin	Nuclear staining
30 s	Distilled water	
10 min	Running lukewarm tap water	Bluing
30 s	Distilled water	
2 min	Eosin Y 1%	Counterstaining
30 s	Distilled water	
30 s	Ethanol 70%	Dehydration
3 min	Ethanol 96%	
3 min	Ethanol absolute I	
3 min	Ethanol absolute II	
3 min	Roti-Histol I	Clearance before mounting
3 min	Roti-Histol II	
	Roti-Histokitt	Mounting

### Statistics

We used estimation statistics and report effect sizes and the confidence intervals for effect sizes. These were assessed using the DABEST toolbox in MATLAB. Bayesian statistics were used for assessing evidence (or lack thereof) for the null hypothesis and for the alternative hypothesis. A Bayesian factor (BF) over three was taken as moderate evidence in favor of the alternative hypothesis. A BF over 10 was considered strong evidence in favor of the alternative hypothesis. A BF of <1/3 was taken as moderate evidence in favor of the null hypothesis, whereas a BF of <1/10 was strong evidence in favor of the null hypothesis. A BF between 3 and 1/3 indicated that the data were ambiguous providing neither evidence supporting the null hypothesis, nor evidence supporting the alternative hypothesis. The lack of evidence could be because of high variability across the samples. Bayesian statistics were calculated in JASP software.

## Results

We compared the effects three water restriction schedules on body weight, Hct, and blood plasma CORT over 80 d. The restriction schedules were “timed,” “continuous,” and “intermittent.” The timed group was given 30 min of *ad libitum* access to water each day. The continuous group received ∼12 ml/d as a single bolus. They began consuming this volume within seconds and, with a few drinking bouts interspersed with food consumption, the entire volume was consumed. If a rat in the continuous group lost >15% of their body weight in a week, then their daily water was increased by 2 ml. Finally, the intermittent group received a repeating schedule of 12 ml of water per day for 5 d, followed by 48 h of *ad libitum* water. On days with volume-limited water access, the consummatory behavior of these rats was noted as similar to that of the continuous group. The choice of volume and timing was based on published literature and a detailed justification can be found in Materials and Methods. A control group was monitored with *ad libitum* access to water for 80 d. Water administration occurred between 2 and 4 P.M. Before water administration, rats were weighed each day and blood was taken from the tail vein twice per week (usually Wednesday and Friday). Measurements were taken before water administration to capture the statuses of the rats in the water restricted state. Each group consisted of six male Sprague Dawley rats. Rats were housed individually to control water intake. All rats were housed in the same room with cages randomly distributed across two racks of individually ventilated cages.

### Rats adapt to water restriction after two weeks and maintain normal Hct levels

**Figure 1.**
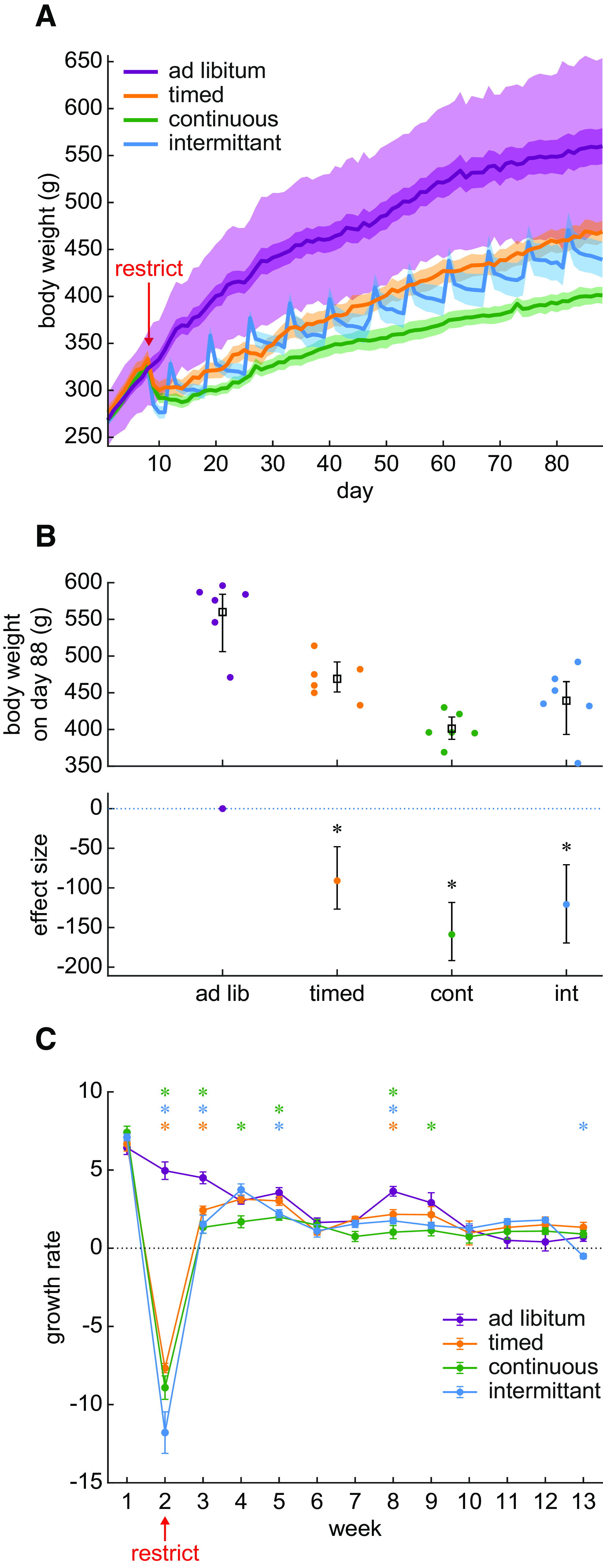
Water restriction evokes an overall body weight reduction that is because of weight loss during the first two weeks. ***A***, Body weight is plotted across the entire duration of the experiment (88 d). Water restriction began on day 8. The mean and SE for each group of rats is shown as a line and shading. The additional light shading around the *ad libitum* group indicates ±2 SDs around the mean. There were six rats per group. ***B***, The final body weight at the end of the experiment was reduced in all water restriction groups relative to the *ad libitum* control group. The upper panel shows the distribution of individual rats (dots) and the group mean and SE. The lower panel shows the effect size relative to the *ad libitum* control group and the error bars show the 95% confidence interval for the effect size. A star symbol indicates that the BF for a post hoc Bayesian t test was greater than 3. ***C***, Weekly body weight change is plotted throughout the experiment. The data markers and lines show the mean and SE for each group of rats. Negative growth (below the dotted line) indicates weight loss, whereas positive points indicate growth. *Post hoc* Bayesian *t* tests on the alternative hypothesis that control group growth was greater than the restricted group’s growth is illustrated with a star when BF is >3. The color of the star indicates the identity of the group being compared against the *ad libitum* control group. A BF > 3 indicates that these data provide evidence supporting the alternative hypothesis that growth in the control group was greater than that of the restricted group. The growth differences occurred primarily during the first two weeks of restriction.

Body weight is frequently used as an indicator of overall health as well as an indirect measure of hydration status in rodents. We compared this measure across the three most frequently used restriction schedules. Figure 1*A* presents the average body weight in each group over 88 d. We plotted ±2 SDs of the *ad libitum* group in light purple shading to facilitate comparison with standard growth curves supplied by animal breeders. The darker shading, as well as the shading around the other groups represents the SE around the mean. Water was removed on day 8. By day 88 (i.e., the 80th day of water restriction), we observed significantly reduced body weights in all water restriction groups relative to the control group (Fig. 1*B*). Body weight in the timed group was reduced by 16.2% relative to the *ad libitum* control group. The effect size and its 95% confidence intervals (ESCI) were at least an 8.5% decrease and at most a 22.5% decrease. Body weight loss in the continuous group was 28.4% (ESCI: between a 21.1% and a 34.3% loss). In the intermittent group, weight loss was 21.6% (ESCI: between a 12.7% and a 30.3% loss). A Bayesian ANOVA suggests that these data provide extremely strong evidence for a difference in weight between restriction schedules (BF = 3866.301). *Post hoc* testing showed that rats on all water restriction schedules lost weight relative to the control group (BFs for continuous, intermittent, and timed were 681.848, 23.841, and 15.394 respective to each group). The weight of rats on the continuous water restriction schedule was also lower than rats on the timed schedule (BF = 31.236). Water restriction clearly effected long-term body weight.

Long-term weight loss may indicate a long-term disruption of rodent health. However, in Figure 1*A*, it appears that much of the weight loss occurs during the first two weeks and that growth normalizes thereafter. It is, therefore, possible that the lower weights after 80 d of restriction were because of a brief period of weight loss occurring at the start of water restriction and that, although these early losses were never re-gained, growth proceeded normally. We formally examined this question by measuring growth as weekly body weight change (Fig. 1*C*). We found that growth largely normalized after two weeks of water restriction. There was an interaction between restriction schedule and week (Bayesian repeated measures ANOVA: BF = 3.198 × 10^118^), which was because of body weight losses that occurred largely in weeks 2 and 3. Thus, our results suggest that the large decrease in body weight after 80 d of chronic water restriction is not because of long-term growth impairment. Instead, since growth normalized after two weeks of restriction, it is likely that this brief window of weight loss is followed by adaptation to the new environmental demands. It is possible however that, before adapting, the weight loss during the first two weeks is because of dehydration.

**Figure 2.**
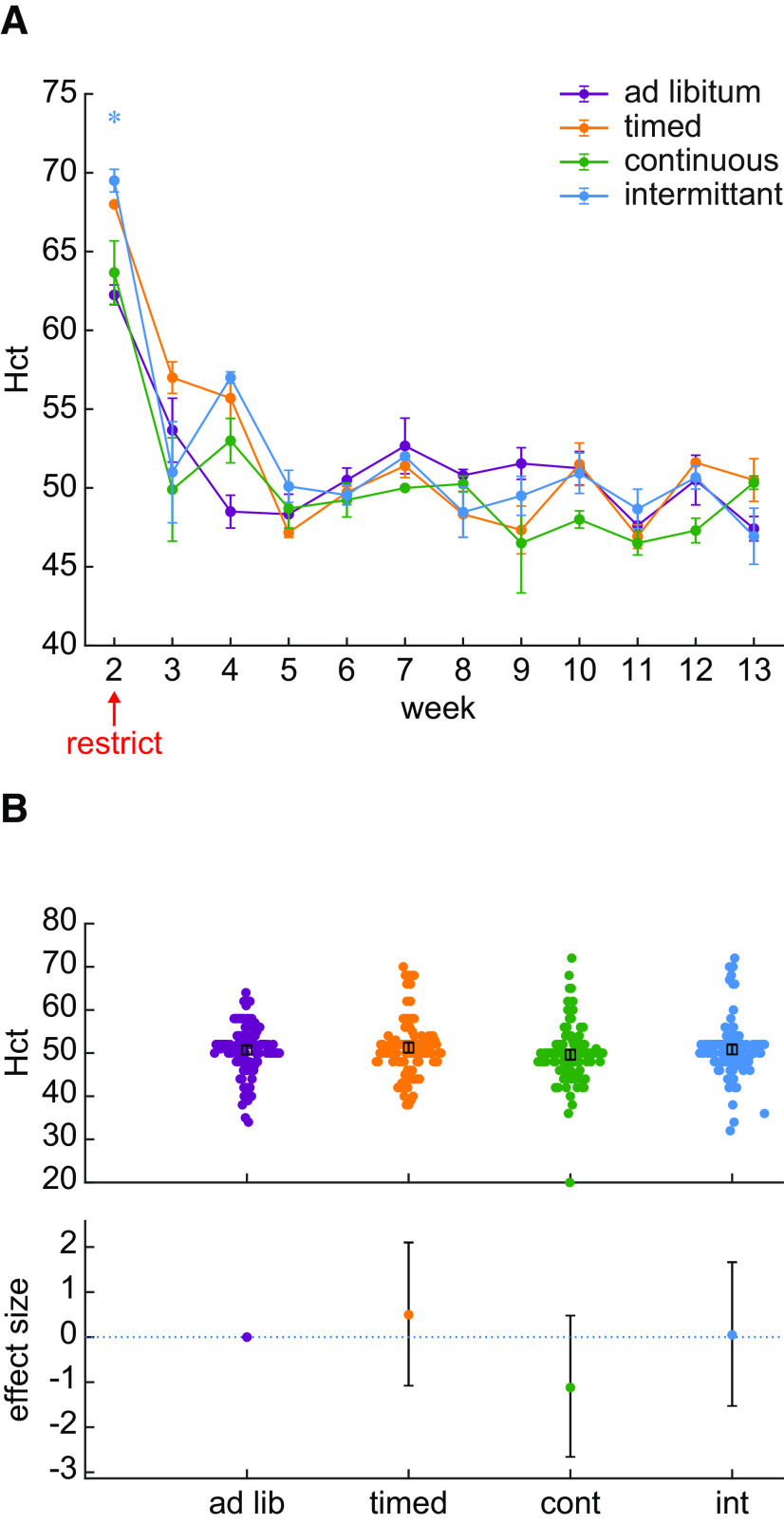
Hct did not differ between the *ad libitum* control group and groups of rats subjected to various water restriction schedules. ***A***, % Hct is plotted as the group average of all samples collected each week. The error bars indicate the SE. Although there in an increase in Hct during the first week of blood collection, this occurred in all groups inclusive of the control group. A star symbol indicates that the BF for a post hoc Bayesian t test was greater than 3. The color of the star indicates the identity of the group being compared against the ad libitum control group. ***B***, An assessment of effect sizes comparing all Hct values collected over 88 d suggests that Hct does not differ between groups. The effect sizes (95% confidence interval) relative to the *ad libitum* control group were: timed group, 1.0% (−2.1 to +4.1%); continuous group, 2.2% (−5.2 to +0.9%); intermittent group, 0.1% (−3.0 to +3.3%).

We used Hct levels to more directly assess whether the first two weeks of water restriction were associated with a change in hydration. Hct was measured as the percent packed cell volume in centrifuged blood samples taken twice per week (Fig. 2*A*). Hct differed over time (Bayesian ANOVA interaction between time and schedule: BF = 1.944 × 10^55^). Hct was increased during the first week of restriction, which was also the first week in which a blood sample was obtained. However, this increase occurred in the control group which suggests that this change was not specific to water restriction. Figure 2*B* shows the Hct values recorded across 80 d of chronic water restriction. The data provide strong evidence supporting the null hypothesis that mean Hct did not differ between restriction schedules (BF = 0.052). Therefore, the drop in body weight during the first two weeks of water restriction is not because of a change in hydration.

### Water restriction evokes a stress response in rats on an intermittent water restriction schedule

**Figure 3.**
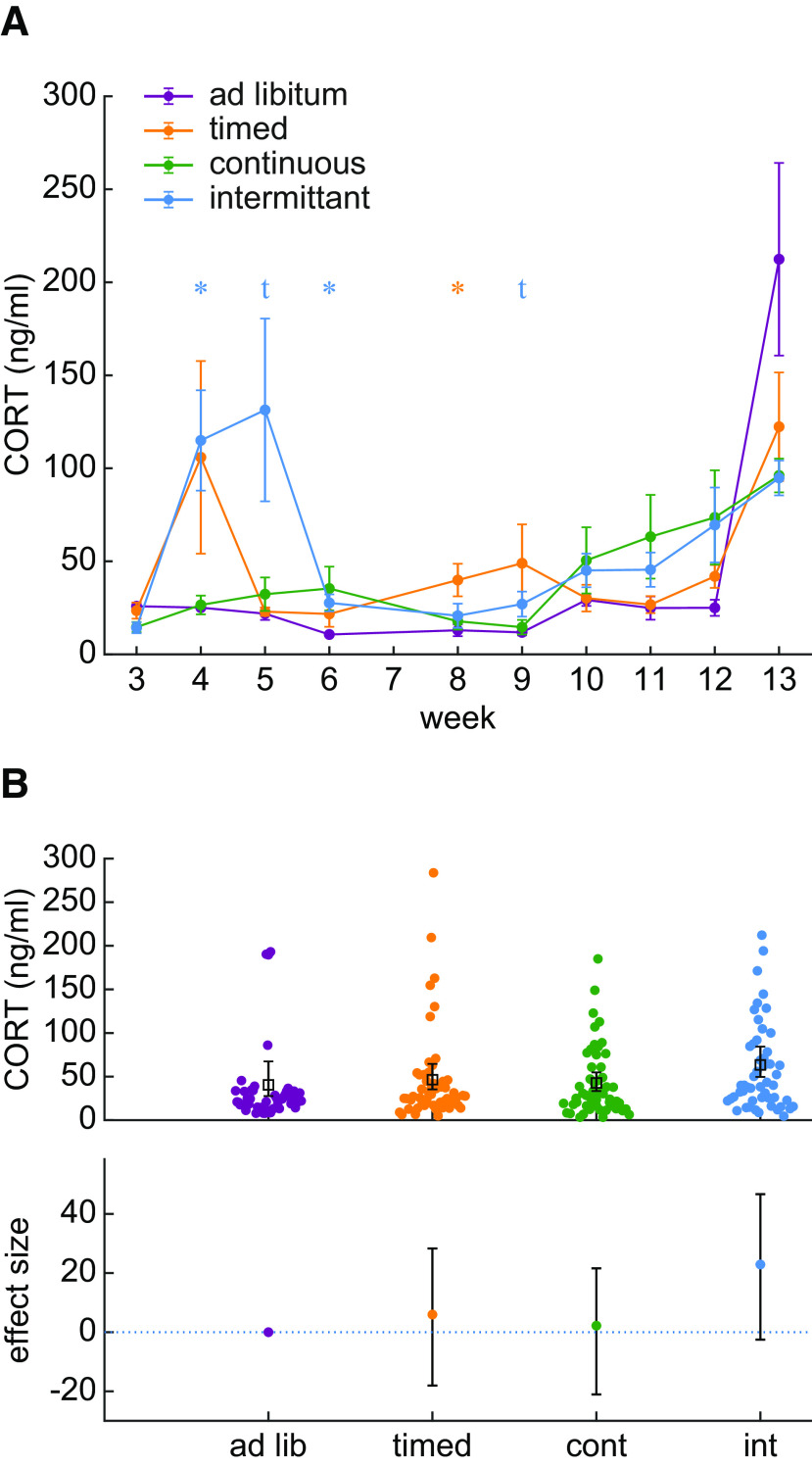
CORT is increased during intermittent water restriction. ***A***, The average and SE of weekly CORT values are plotted for each group of rats. The intermittent group has occasionally elevations in CORT. A star symbol indicates that the BF for a post hoc Bayesian t test was greater than 3. The t symbol indicates a BF value greater than 2. The color of the symbols indicate the identity of the group being compared against the ad libitum control group. ***B***, Collapsing all measurements over time revealed that the intermittent group has elevated CORT relative to the *ad libitum* control group. The upper panel shows individual data points, and the lower panel shows the effect sizes of group differences relative to the *ad libitum* control group.

Adaptation to water restriction may increase circulating stress hormones, given that food restriction can elevate CORT in rodents (Abrahamsen et al., 1995; Heiderstadt et al., 2000a). We measured blood plasma CORT twice per week (Fig. 3*A*). CORT values are reported starting from week 3 because inadequate blood volumes were obtained in week 1 (before restriction) and week 2 (start of restriction). There was an interaction between restriction schedule and time (BF = 1.353 × 10^13^), which were driven by an early increase in the intermittent group (*post hoc* Bayesian *t* tests, BF > 3, except BF = 2.082 for week 5 and BF = 2.423 for week 9). Given that week 3 was associated with reduced growth (see Fig. 1*C*) in all restriction groups, the specificity of the stress response for the intermittent restriction group suggests that an initial adaptation during the first two weeks of water restriction is not associated with a stress response.

On the other hand, a stress response may also be evoked by environmental instability which occurs specifically in the intermittent group. Environmental instability occurs in the intermittent group because these rats repeatedly encountered water losses after the periodic 2-d breaks from restriction. The instability in the environment altered the body state of these rats by producing a highly variable “saw-tooth” pattern in intermittent group body weights (see Fig. 1*A*). Our data suggest that the environmental instability encountered by the intermittent schedule group could be a psychological stressor that evokes a chronic stress response. We assessed this by collapsing CORT measurements from all timepoints to assess whether CORT was overall higher in the intermittent group (Fig. 3*B*). The ESCI in the timed group spanned from a 46% reduction to a 69.4% elevation in CORT, which suggests no effect of timed restriction on CORT. Similarly, the continuous group ESCI ranged from a 51.8% reduction in CORT up to a 53.0% increase in CORT. However, the intermittent group was associated with an effect size of 56.6% with the ESCI ranging from roughly no change (−6.9%) up to a 115.0% increase. CORT in the *ad libitum* control group was 40 ± 9 ng/ml, whereas in the intermittent group it increased to 63 ± 9 ng/ml. Given that the ESCI is the 95% confidence interval for the effect size, the average CORT was likely increased specifically in the intermittent group. We formally tested the alternative hypothesis that the intermittent group had higher CORT than the *ad libitum* control group using a Bayesian Mann–Whitney *U* test (because of the skew of the intermittent group distribution). A BF of 27.47 suggested that the collected data may be taken as strong evidence in favor of the alternative hypothesis. Therefore, the intermittent water restriction schedule used here may present a psychological stressor that evokes a significant increase in CORT that is around 56.6% higher than under *ad libitum* conditions.

### Water restriction is not associated with adaptation of the Loops of Henle

**Figure 4.**
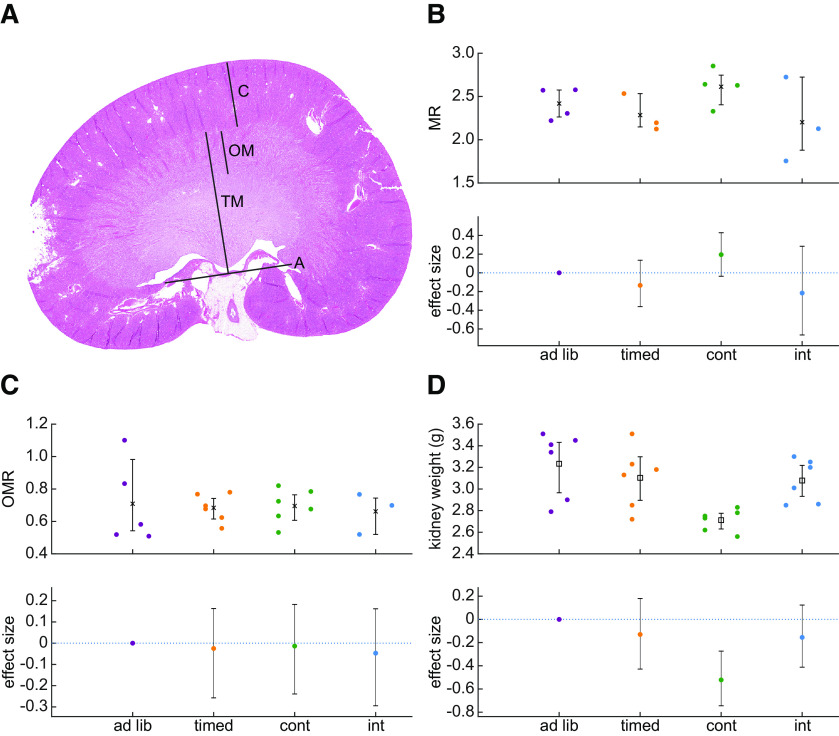
Water restriction is not associated with alterations in the Loops of Henle. ***A***, This example tissue section of the kidney shows the gross anatomic makers used to delineate the cortex (***C***), the outer medulla (OM), the total medulla (TM) demarcated as the distance between the capsule and the “assisting line” (***A***). The assisting line connects the two points where the ureter connects to the kidney and was used to have a standardized starting point for measurements. The TM may or may not include the entire inner medulla, which sometimes crossed the assisting line. We assessed RMT using two formulae. The first was the OM to C ratio (OMR) and the other was the TM to C ratio (MR). ***B***, ***C***, Neither the MR (***B***) nor the OMR (***C***) differed across groups of rats. The plots show the individual data points where complete sections could be obtained to make a clear assessment of these measures. The lower panel shows the effect sizes relative to the *ad libitum* control group and the 95% confidence intervals of those effect sizes. ***D***, Postmortem kidney weight was measured and indicated a reduction in kidney weight in the group of rats subjected to continuous water restriction. The upper panel shows individual data points and the lower panel shows effect sizes and confidence intervals relative to the *ad libitum* control group.

In response to water scarcity, organisms adapt by producing hyperosmotic urine. This physiological adaptation depends on the lengths of the Loops of Henle in the renal medulla (Schmidt-Nielsen and O’Dell, 1961). There is evidence that structural adaptation of the Loops of Henle can occur over the timescale of a few weeks during water deprivation (Trinh–Trang–Tan et al., 1987). We assessed whether the water restriction schedules used here were severe enough to promote structural changes to the kidneys using postmortem histologic measurements of relative medullary thickness (RMT). RMT is indicative of a shift in the size of the renal medulla relative to the cortex indicating lengthening of the Loops of Henle (Sperber, 1944). An increased RMT is associated with urine osmolality and can therefore be used as a surrogate measure of an organism’s ability to produce hyperosmotic urine in response to water scarcity (Schmidt-Nielsen and O’Dell, 1961; Heisinger and Breitenbach, 1969; Brownfield and Wunder, 1976). Kidney sections were inspected, and measurements made by an individual blind to the water restriction group assignments of the rats. RMT was measured according to two formulae that capture changes in the size of the renal medulla relative to the rest of the kidney (Fig. 4*A*). We found that neither of these measures differed across groups (Fig. 4*B*,*C*). A Bayesian one-way ANOVA suggested moderate support for the null hypothesis for similar outer medulla to cortex ratio (OMR) across groups (BF = 0.23). The result of the Bayesian one-way ANOVA for total medulla to cortex ratio (MR) data were ambiguous, but close to the threshold for moderate evidence supporting the null hypothesis (BF = 0.66). Taken together, it is unlikely that water restriction schedules evoked lengthening of the Loops of Henle. Interestingly, there was a significant difference in postmortem kidney weight across groups (Bayesian one-way ANOVA, BF = 7.514). The data suggest that, in the continuously restricted group, kidney weight was reduced (Fig. 4*D*). The continuous group kidney weight was 16.1% lower than the kidneys in the *ad libitum* control group with a 95% confidence interval of an 8.5% loss up to a 23.0% loss. *Post hoc* Bayesian *t* tests indicated that the kidney weights of rats in the continuous water restriction group were significantly lower than those of the *ad libitum* group (BF = 13.08), as well as the intermittent water restriction group (BF = 14.35) and the timed group (BF = 5.377). Collectively, these data suggest that water restriction is not severe enough to evoke structural adaptations of the renal medulla; however, continuous water restriction may lead to a modification of gross kidney mass.

## Discussion

Water restriction is a widely used tool in neuroscience research in rodents; however, effects of these water restriction schedules on objective measures of hydration and on stress hormone level are unknown. It is also possible that rodents readily adapt to water restriction by structural modification of the kidneys. Here, we measured daily body weight and bi-weekly plasma CORT and packed red blood cell volume (Hct) over 80 d in four groups of male rats subjected to different water restriction schedules that are commonly used in behavioral studies. We observed a one- to two-week period in which body weight is diminished in all three restriction groups and followed by normal growth. The reason for the weight loss during the initial two weeks of water restriction may be a mild anorexic response that reduces water loss through feces (Watts, 1999; Desai et al., 2005; Rowland, 2007; Bekkevold et al., 2013). Indeed, rats reduce their *ad libitum* food intake during water deprivation (Bealer et al., 1983). We found no evidence for changes in Hct, suggesting that hydration remained normal and that rats readily adapt to the water restriction schedules used in the present study. As part of this adaptive response, the renal mechanisms for water conservation may be engaged so that rats can resume normal growth (despite limited water availability) beginning in the third week of restriction. Kidney histology was used to measure changes in the renal medulla and demonstrated that these commonly used restriction schedules are not severe enough to drive long-term adaptation of the renal system. However, we cannot exclude the possibility that renal aquaporin expression may have adapted in response to water restriction, given that plasticity of aquaporin expression has been demonstrated in the response of rodents to changes in seasonal water scarcity in the wild (Gallardo et al., 2005). Although stress hormones were also not generally altered by water restriction, the intermittent group had a minor elevation in CORT that may be a behaviorally and neurobiologically-relevant stress response. Overall, we found that months-long use of timed and continuous restriction schedules in rats maintains their welfare, but that intermittent restriction evokes a minor stress response.

Our results were obtained in male rats; however, it is important to recognize that these results may not generalize to other rodents, such as mice, and may also differ between males and females. Although little work has been done in female rodents, prior work has compared the physiological responses of rats and mice to brief periods (e.g., 24 h) of complete water deprivation. A review of these studies on total deprivation (Rowland, 2007) has shown that dehydration-related anorexia and blood plasma osmolality changes are two times stronger in male mice compared with male rats. It is not possible to extrapolate from brief and complete deprivation to the effects of chronic water restriction; however, the possibility that rats and mice respond differently should be considered by researchers using mice in behavioral neuroscience research.

### Implications for rodent welfare

The welfare of rodents is a key objective in all experiments primarily for ethical reasons, but also because unhealthy animals cannot yield normal data. Water restriction could affect welfare by evoking dehydration; however, our results suggest that the restriction schedules used here do not cause dehydration. We assessed hydration by measuring Hct. Typical Hct values in adult, male rats have been reported to range between 33 and 57 (Houtmeyers et al., 2016) and around 42 in rats that are not water restricted (Fitzsimons, 1963). Although Hct depends on age and body weight, it largely stabilizes between 40 and 45 in rats of the age and body weight used in the present study (Belcher and Harriss, 1957). Therefore, we observed normal Hct values that are typical for non-water restricted rats of this gender and age. Our findings suggest that hydration is not affected by any of the water restriction scheduled used in the present study. Normal hydration is presumably maintained by the rats via renal adaptation and the production of hyperosmotic urine. Importantly, welfare may be negatively impacted by a stress response, which was observed only in the rats subjected to an intermittent restriction schedule.

### Implications for maintaining stable motivation of rodents during goal-directed behavioral tasks

The schedules tested here are commonly used in rodent behavioral neuroscience experiments. For example, some laboratories have chosen to use continuous volume-limited restriction because motivation is reduced after each break in an intermittent schedule (Busse et al., 2011; Carandini and Churchland, 2013; Guo et al., 2014). However, intermittent schedules are also common (Schwarz et al., 2010; Histed et al., 2012; Guo et al., 2014). Motivation can also be maintained at a stable level with a timed access schedule. Various laboratories have motivated behavior by time-limited water access from 10 min/d to 1 h/d (Barnet et al., 1997; Denniston et al., 1998; Laraway et al., 2003; Scott et al., 2013; Foo et al., 2021). Our finding that body growth is temporarily reduced for the initial approximately two weeks of water restriction for all schedule types suggests that this is a period of adaptation. During this period when rodents adapt to the new environmental constraints, they may have increased motivation to collect reward. Our results suggest that collection of behavioral and neurobiological data during this period may include instabilities in how rodents respond to water rewards. Therefore, allowing this adaptation period to pass before collecting data may be a useful practice in behavioral neuroscience research.

Other recent methods of water restriction that provide less palatable 2% citric acid water *ad libitum* in the home cage have not been assessed for stress hormone release or kidney adaptation (Reinagel, 2018; Urai et al., 2021). However, it is likely that our findings would be similar under those conditions because rodents given citric acid in water effectively self-restrict their water intake (because of the aversive taste of the water) to approximately the same volume of daily water provided in our study to the continuous restriction and intermittent restriction groups (Reinagel, 2018). Although providing *ad libitum* citric acid water is less labor intensive and is an efficient way to motivate rodents without needing to administer precise daily allotments of water tailored to individual rats, the use of citric acid results in fewer daily trials performed compared with water restriction (Reinagel, 2018). Therefore, the water restriction schedules used here may be most relevant to studies that aim to maximize the number of trials performed.

### Implications of the stress response in rats on an intermittent water restriction schedule

Intermittent water restriction could be a physiological stressor because of the saw-tooth pattern of repeated weight loss and weight rebound. It could also be a psychological stressor because of unstable environmental water availability. We observed a mean increase of plasma CORT to 63 ng/ml in rats on the intermittent restriction schedule. The blood plasma collection procedure (brief single tail vein puncture requiring <15 s) did not affect the measurements because CORT requires ∼20 min to elevate after a tail puncture (Haemisch et al., 1999). Our data support the notion that intermittent water restriction is a stressor, but it is a relatively minor stressor compared with air puff startle (450 ng/ml; Engelmann et al., 1996), restraint stress (250–800 ng/ml; Plotsky and Meaney, 1993; Akana and Dallman, 1997) and forced swimming (400–500 ng/ml; Armario et al., 1986). However, CORT level during an intermittent water restriction schedule is similar to the CORT increase after handling in rats subjected to early life maternal separation stress (100 ng/ml; Kalinichev et al., 2002) and the stress response to environmental noise (100–200 ng/ml; Armario et al., 1986). In sum, our results demonostrate that the water restriction schedules used in this study provided appropriate levels of hydration, but that rats on an intermittent water restriction schedule have a stress response which may have behavioral, neurochemical, and neurophysiological effects.

## Synthesis

Reviewing Editor: Laura Bradfield, University of Technology Sydney Decisions are customarily a result of the Reviewing Editor and the peer reviewers coming together and discussing their recommendations until a consensus is reached. When revisions are invited, a fact-based synthesis statement explaining their decision and outlining what is needed to prepare a revision will be listed below. The following reviewer(s) agreed to reveal their identity: Cornelius Schwarz, Robert Rodgers.

The manuscript provides important new information about the physiological effects of water restriction schedules. This is important because water restriction is a commonly used technique amongst many laboratories who should be aware of any unintended side effects.

Both reviewers found the manuscript to be well written, solidly realised, and addressing an important question. Both also had a few queries to be answered as detailed in the reviews below. Please include additional data to answer the queries of reviewer #1 if possible. In addition, as only male rats were used, could the authors please add this information to the abstract, and a statement to the discussion regarding the fact that these results may not generalise to females.

The specific reviews are as follows:

Reviewer #1

This study pursued a worthy aim, to quantify physiological effects of different water restriction schedules in rats often used in behavioral and (neuro-)physiological studies. Such data will help researchers to decide which restriction schedule is most appropriate for their question, and to assess whether expected effects of restriction may have impact on their data.

The authors first assessed the body weight under the restriction schedules and found that all schedules led to decreased weight compared to control. Importantly, however, this was due to effects impacting within the first two weeks of restriction. Thereafter the growth rate was normal.

Haematocrit measurements did not support the notion of dehydration with any of the schedules. The reduced weight is therefore likely based on decreased food intake (to save water contained in the feces). After that the growth rate suggests that the strategy of reduced food intake is abandoned likely enabled by onset of renal adaptation.

CORT levels were normal (except the intermittent group’s first few weeks of restriction). The authors speculate that the intermittent group suffers environmental instability (change from restriction to free water leading to oscillations in weight) which causes a stress response.

Adaptation to the restriction schedules is not signaled by the estimated length of the renal loop of Henle. However, a reduction of gross kidney volume was found in the continuous group.

I think this study is well done and I recommend its publication. I have a few comments:

1) The transient ‘anorexia response’ is an assumption carried by transient weight loss (lack of gain). Did the authors directly measure food intake to further support this notion? Or did they measure the composition of feces around the time of restoring growth rate?

2) Could you provide the weight curves with 2*std error ranges? This would allow to assess in how far the weight of an experimental animal is within the ‘normal’ weight range.

3) Similarly, the onset of renal adaptation after the initial weight loss (or lack of gain) is an assumption. Are there data to support this notion further (urine volume, urine composition, etc.)

4) The assumed renal adaptation is not supported by the histological estimation of the Henle loop’s length. Please discuss which other adaptation processes could play a role (that would not affect the loop’s length).

5) I find the reduction of kidney volume/mass with continuous restriction puzzling. What are possible reasons for this phenomenon?

6) Does the finding that CORT levels are unchanged in timed and continuous groups argue against the assumption of an anorectic response?

7) Please discuss the estimated size and duration of a CORT elevation due to taking blood.

8) There is a huge and growing community working on mouse models. Please discuss your results in terms of possible impact on mouse work.

Reviewer #2

Although water restriction schedules have been used in behavioural research for many years, their effects on animal welfare have only been studied at short fixed timepoints. Head-fixed methodologies, where behaviour is water-reinforced, require water restriction over much longer timeframes. Hence, the present study examined the effects, longitudinally over 80 days, of 3 different restriction schedules. The dependent measures were bodyweight gain, hydration, plasma CORT and renal adaptation. The results convincingly show that, following the initial 2 week period, rats readily adapt to these new schedules (as assessed by bodyweight, hydration and renal structure/function). The one exception concerns the intermittent restriction condition where there is a significant increase in plasma CORT over weeks 3-5. This suggests a modest stress reaction which may confound interpretation of the effects of experimental manipulations. Overall, these findings provide considerable reassurance for those using water deprivation to motivate behavioural responses in a wide variety of research contexts.

The manuscript is well-written, with an excellent literature review, detailed methodology, as well as clear analysis, presentation and discussion of results. I have very few critical comments.

1. The authors need to be more careful in the Abstract and elsewhere in stating that ‘the present water restriction schedules maintain animal welfare’. The significant CORT response in the intermittent restriction condition is at odds with this statement.

2. Methods. Subjects. For replication purposes, it would be helpful to include the age/starting weight range of the animals as well as the dimensions of their home cages.

3. Results. There is no mention of the apparent large increase in plasma CORT levels seen in the ad lib condition in week 13?

p12, line 295 - palatable?

## Author Response

Dear Dr. Bradfield, Dear Reviewers:

We appreciate the time that you and each of the Reviewers have taken to consider our manuscript. We believe that your suggestions have improved the paper. Below, we provide a specific point-by-point reply to each of the Reviewers. The Reviewers’ text is in black and our response is in blue text. In the text, we have re-written and modified the writing (bolded blue text) according to the suggestions that were kindly provided by the Reviewers. We agree with the synthesis statement that these results do not generalize to females and we have added this important statement to the discussion section as requested (please see lines 286 - 295). The information about the use of males in this study is in the abstract in the initial submission (line 7), so we did not make additional modifications to the abstract. Please let me know if the suggestion was to add this information to another location in the abstract.

Thank you again for taking time to review our work and help us to improve it.

Reviewer #1

1) The transient ‘anorexia response’ is an assumption carried by transient weight loss (lack of gain). Did the authors directly measure food intake to further support this notion? Or did they measure the composition of feces around the time of restoring growth rate?

We agree with the Reviewer’s suggestion that these data would lend support to the claim that the transient weight loss observed in our work is due to the anorexic response (see references 34 to 37 in paper). Unfortunately, we did not collect either of the data sets suggested by the Reviewer.

2) Could you provide the weight curves with 2*std error ranges? This would allow to assess in how far the weight of an experimental animal is within the ‘normal’ weight range.

We would like to retain the current plot showing only the SEM for consistency throughout the manuscript. However, we have included here the plot of 2*SE for the Reviewer. It is clear that none of the restriction schedules overlap with the ‘normal’ range (i.e., the ad libitum group). We are open to including the plot if the Reviewer views its inclusion as a critical addition to the paper.

3) Similarly, the onset of renal adaptation after the initial weight loss (or lack of gain) is an assumption. Are there data to support this notion further (urine volume, urine composition, etc.)

We agree that measuring urine osmolality would support the claim of renal adaptation. At least the measure we used, RMT, is associated with urine osmolality and can therefore be used as a surrogate measure (see lines 215 - 217).

In response to the Reviewer’s concerns (both in question #1 and in this question #3), we have removed from the results all speculation about the anorexic response and renal adaptation relative to the early weight loss. These removals are strike-throughs on lines: 152-156, 168, 175-177. We now only address these ideas in the discussion section. We also cited an additional study showing that rats reduce their food intake during water restriction. Please see lines 258 - 286 of the discussion section to see how this text has been incorporated into the discussion section. We also reorganized that paragraph to improve its flow with the addition of new information, so some existing lines were copied and moved. Their original location of moved text was struck through.

4) The assumed renal adaptation is not supported by the histological estimation of the Henle loop’s length. Please discuss which other adaptation processes could play a role (that would not affect the loop’s length).

We think that one other potential mechanism is changes in aquaporin expression. (Unfortunately, we cannot test this without new experiments). We discussed this possibility in the discussion section and it has moved to lines 268 to 270.

5) I find the reduction of kidney volume/mass with continuous restriction puzzling. What are possible reasons for this phenomenon?

We also find the reduction in kidney mass surprising. We initially expected that it was a sign of renal adaptation and expected to observe a change in RMT in the histology; however, there was no change in RMT. We have looked into the literature and also spoke with veterinarians but have been unable to find an explanation for this observation.

6) Does the finding that CORT levels are unchanged in timed and continuous groups argue against the assumption of an anorectic response?

The anorexic response is a natural adaptation to water scarcity. Therefore, we think that it should occur regardless of whether there is a stress response or not. The anorexic response has been demonstrated in the prior literature and is reviewed in Rowland (2007), which is citation #35 in the resubmitted manuscript.

7) Please discuss the estimated size and duration of a CORT elevation due to taking blood.

Please see lines 325 - 327 and citation showing that the CORT increase due to tail puncture occurs 20 min after puncture and cannot be observed in our procedure, which required <15 sec.

8) There is a huge and growing community working on mouse models. Please discuss your results in terms of possible impact on mouse work.

We have added discussion of this topic in lines 286 - 295.

Reviewer #2:

1. The authors need to be more careful in the Abstract and elsewhere in stating that ‘the present water restriction schedules maintain animal welfare’. The significant CORT response in the intermittent restriction condition is at odds with this statement.

We agree and have noted that the maintenance of welfare is in the timed and continuous restriction groups, but that the intermittent group had a stress response. We have removed line 14 from the abstract. We have updated the following lines: 72 - 75, 283 - 285, 309 - 310.

2. Methods. Subjects. For replication purposes, it would be helpful to include the age/starting weight range of the animals as well as the dimensions of their home cages.

Thank you for this suggestion. We have added the weight (from which post-natal age can be calculated). Please see line 353. We have also added the cage information. Please see lines 354 - 355.

3. Results. There is no mention of the apparent large increase in plasma CORT levels seen in the ad lib condition in week 13?

Thank you for your question. We found that all groups had an increase in the final level. The increase in the ad libitum group was not significant compared to the other groups and had extremely large variability.

p12, line 295 - palatable?

Thank you for indicating this typographical error. We have corrected it.
